# Local Conditions and Environmental Gradients Shape the Abundance and Size Structure of a Non‐Native Intertidal Species in Atlantic North America

**DOI:** 10.1002/ece3.72532

**Published:** 2025-12-12

**Authors:** Giuseppe Garlaschè, Bernardo R. Broitman, Cyrena Riley, David Drolet, Kimberly L. Howland, Christopher W. McKindsey, Piero Calosi

**Affiliations:** ^1^ Laboratoire de Physiologie Écologique et Évolutive Marine, Département de Biologie, Chimie et géographie Université du Québec à Rimouski Rimouski Quebec Canada; ^2^ Québec‐Océan Laval University Quebec Quebec Canada; ^3^ Fisheries and Oceans Canada Maurice Lamontagne Institute Mont‐Joli Quebec Canada; ^4^ Departamento de Ciencias, Facultad de Artes Liberales Universidad Adolfo Ibáñez Viña del Mar Chile; ^5^ Fisheries and Marine Mammal Science Division, Arctic Region Fisheries and Oceans Canada Winnipeg Manitoba Canada

**Keywords:** environmental gradients, intertidal, macroecology, non‐native species, population dynamics, range shifts

## Abstract

Variation in abundance and size along environmental gradients provides insights into the capacity of species to adapt to changing environmental conditions and predict range expansions, this being particularly relevant for non‐native species. Here, we evaluate how population density, shell height, and size structure vary in relation to environmental factors across a broad latitudinal gradient in the non‐native *Littorina littorea* . We sampled ten locations, covering nearly the entire range of *L. littorea* in North America and measured snail density, shell height, substrate rugosity and algal biomass. We extracted other environmental factors from satellite data and used them to assess the influence of local characteristics and environmental gradients on mean snail density, mean shell height and size structure. We found evidence of a weak positive relationship between snail density and rugosity. Mean shell height showed a strong positive relationship with growing season length and a negative relationship with air temperature. We also found that high and variable temperatures during summer, length of the growing season, and high rugosity, negatively impacted the frequency of small individuals. Instead, high water temperature during the spawning period, low temperature variability during summer, and low substrate rugosity are positively associated with a higher frequency of small individuals. Our results indicated that substrate rugosity positively influenced the abundance of this non‐native littorinid independent of the climatic conditions experienced. Instead, variation in shell height largely reflected the environmental gradient found throughout its range. In particular, we argue that the lower abundance of small snails in sites with high and variable summer temperatures reflects increased juvenile mortality, while the higher frequency of small snails in sites with warmer spawning periods and more stable summers indicates enhanced recruitment and population growth. These findings support the potential for *L. littorea* to expand its range poleward with the progression of climate warming.

## Graphical abstract

We investigated how density and body size of the non‐native intertidal snail *Littorina littorea* vary along a 10° latitudinal gradient (> 1700 km) in North America. Our analyses show that local substrate complexity drives density patterns, whereas body size is shaped by climatic factors, with higher and more variable summer temperatures reducing juvenile survival and warmer, stable spawning conditions promoting recruitment. These results suggest that climate warming will facilitate northward expansion of *L. littorea* while contributing to population declines at southern sites.

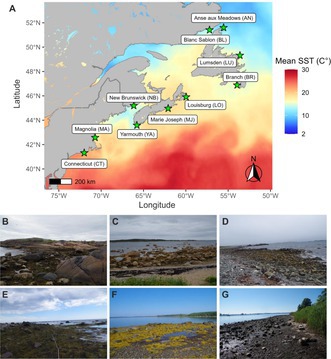

## 1 Introduction

Non‐native species (NNS) are rapidly shifting their geographic ranges and threatening global ecosystem structure and functioning (Molnar et al. 2008; Gallardo et al. 2016). Currently, the impact of NNS on ecological systems is exacerbated by the co‐occurrence of global changes, which are reshaping habitats, facilitating dispersal and creating new introduction mechanisms (Hellmann et al. 2008; Mainka and Howard 2010). In turn, climate change is causing range expansions and shifts for previously introduced species, potentially enhancing their chances to thrive in formerly unsuitable regions (Hellmann et al. 2008; Moran and Alexander 2014; Lawlor et al. 2024). In this context, understanding the population dynamics of non‐native species may help us characterize their capacity to adapt to new environmental conditions, as predicting their eventual range expansion has become a pressing challenge (Brzeziński et al. 2019; Seebens et al. 2019). Studies to date suggest NNS often benefit disproportionately (relative to native species) from climate change, which promotes range expansions, usually toward higher latitudes (Neumann et al. 2013; King et al. 2021; Bradley et al. 2024).

Multiple environmental factors may influence the biogeography and population structure of a species in space; however, temperature plays a paramount role for ectotherms (Hutchins 1947; Brown et al. 2004). Intertidal communities are particularly exposed to extreme thermal variation and thus are expected to be strongly influenced by temperature (Helmuth et al. 2006; Bozinovic et al. 2011). Intertidal organisms from different latitudes and thermal regimes exhibit variation in population structure, which is reflected by differences in abundance and body size (Jaramillo et al. 2017; Oróstica et al. 2020). For body size in particular, Bergmann's rule (Bergmann 1848) predicts larger body sizes in colder climates. This rule was originally formulated based on homeotherms but has been observed in many ectothermic intertidal species (Lee and Boulding 2010; Jaramillo et al. 2017; Johnson et al. 2019; Hernández‐P et al. 2023). In intertidal organisms, this pattern may be due to mechanisms such as compensation for colder temperature regimes in the north and/or biotic factors such as predation and food supply (Lee and Boulding 2010; Jaramillo et al. 2017; Hernández‐P et al. 2023).

Multiple factors beyond temperature may drive differences in population structure along a latitudinal gradient, including seasonality and climatic variability, as well as primary production, and thus food supply that changes from high to low latitude (Helmuth et al. 2002; De Frenne et al. 2013; Huston and Wolverton 2009; Chavez et al. 2011). Likewise, local factors, including substrate complexity, may strongly influence rocky shore intertidal communities, shaping population structure independent of latitudinal effects (Kohn and Leviten 1976; Suzanne Witte et al. 2025). In this context, using only latitude as a discriminant may lead to imprecise predictions and interpretations of the role of multiple environmental processes as drivers of NNS distributions (McDowell et al. 2014; Hadiyanto et al. 2024).

Our study examined changes in population density and shell height of an intertidal NNS, the common periwinkle *Littorina littorea* (Linnaeus, 1758), along a large latitudinal gradient in its introduced geographic range in North America. In particular, we focused on determining which environmental factors varying along the latitudinal gradient investigated were more strongly associated with population density and shell height. *Littorina littorea* is an ideal study species to address our aim as (1) it is an intertidal non‐native species in North America (Chapman et al. 2007), originally from Western Europe, having experienced relatively recent and rapid range expansions (likely between 1860 and 1890) along the Atlantic coast (Chapman et al. 2007), and more recently (in the last 15 years), in the St‐Lawrence Estuary (Diéval et al. 2011; Drolet D., personal observation 2025). The non‐native context makes the species a compelling case for studying rapid adaptation over a short evolutionary timescale. In addition, (2) this species' current North American distribution spans 13 degrees of latitude and various climatic zones, and it is considered an ecologically dominant species of intertidal rocky shores in the region (Reid 1996). Unlike other North Atlantic periwinkles, *L. littorea* has a pelagic larval stage and is an omnivorous grazer not limited to a specific substrate type, which allows it to rapidly thrive in a wide range of habitats (Reid 1996). Finally, (3) *L. littorea* is predicted to expand northward beyond its current distribution limits as a consequence of global changes (Goldsmit et al. 2018). We thus sampled 10 locations along the Atlantic coast of North America (covering 10° of latitude and about 1700 km) to obtain data on the species' population density, shell height, and local ecological characteristics such as substrate complexity and algal biomass. We extracted thermal environmental factors for each location through satellite measurements and used them, together with local factors such as algal biomass and substrate rugosity, to study their influence on snail density and shell height patterns. We investigated changes in shell height evaluating both mean shell height differences and size structure shifts among locations. Although previous studies have investigated intertidal communities' body size and density patterns along latitudinal gradients, few focus on the differential influence of multiple environmental factors on the observed trends of variation. Our approach addresses the influence of various environmental factors along a latitudinal gradient, beyond the effect of latitude alone, offering a more comprehensive and nuanced understanding of the environmental drivers of density and size geographic variation in intertidal organisms.

## 2 Materials and Methods

### 2.1 Sampling Region and Design

**FIGURE 1:**
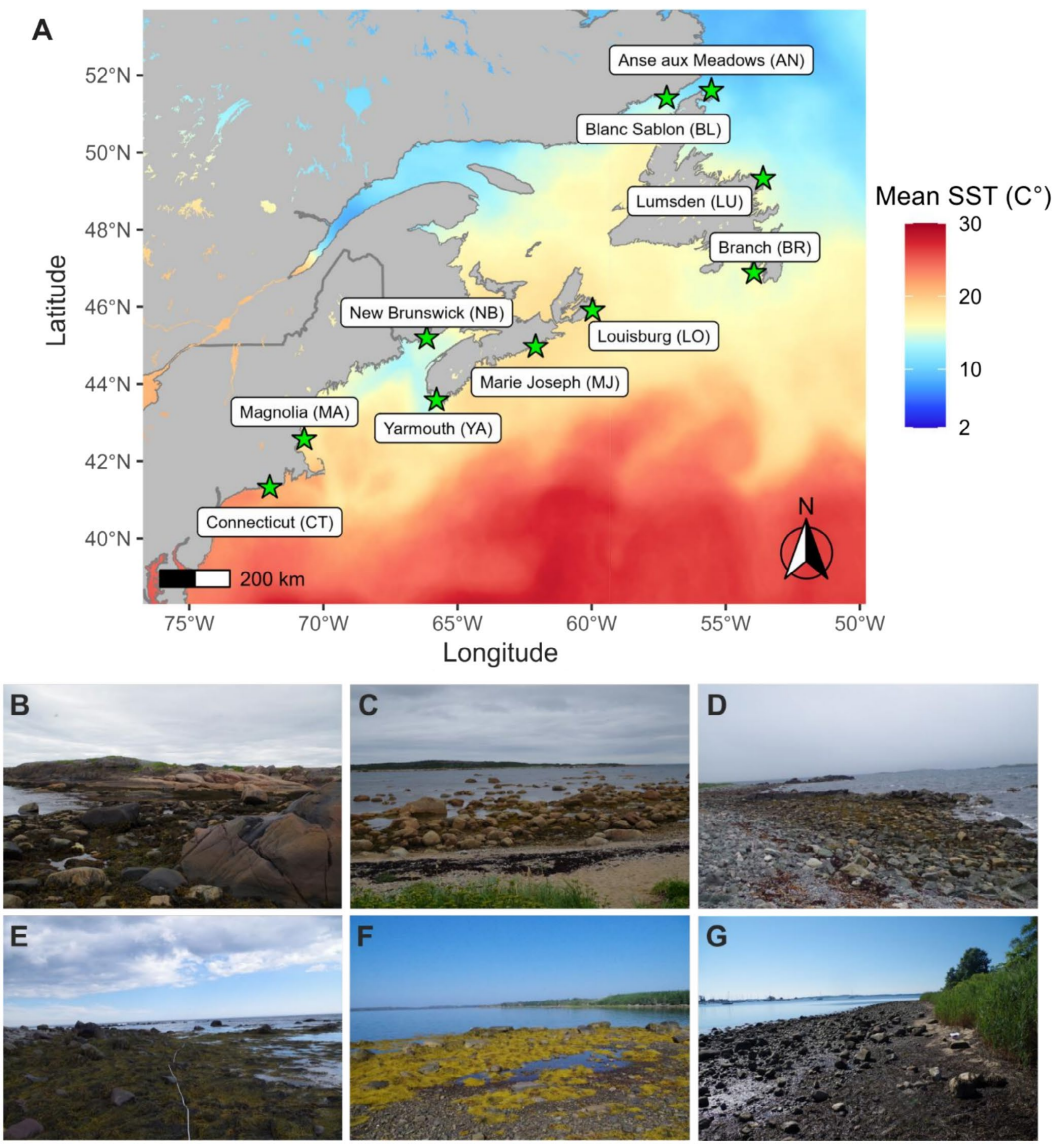
Map (A) and pictures (B–G) of sampling sites where adults of the common periwinkle snail *Littorina littorea* were collected along its geographical distribution range on the Atlantic coast of North America and mean sea surface temperature (SST), a monthly composite of summer 2022 Multi‐scale Ultra‐high Resolution grid data (*Data Source:* NOAA ERD and CoastWatch West Coast Regional Node (Dataset ID: jplMURSST41mday)). The name of each location is given, together with the acronym used in the main text in brackets. Pictures of sampling locations, provided as habitat examples, represent: Blanc Sablon (B), Lumsden (C), Louisburg (D), Branch (E), Marie Joseph (F), Connecticut (G).

Ten rocky shore locations (Figure 1, Table S1) along the North America Atlantic coastline were sampled between July and September 2022, covering a latitudinal range of 10° and approx. 1700 km: from Esker Point Beach (CT, USA; 41° N) to Anse aux Meadows (NL, Canada; 51° N). This enabled us to include almost the entire (~83%) North American distribution range of *L. littorea* (Reid 1996; Chapman et al. 2007). We chose locations to comprehensively cover the range of latitudes investigated, with at least one location sampled within each 2° N of latitude. Site selection was further guided by the presence of rocky shores with reasonably similar characteristics and confirmed occurrence of the target species. In addition, we explored locations at higher latitudes potentially occupied by the species since the last documented survey (Blakeslee et al. 2021), to check for any evidence of expansion with the progression of ocean warming (Goldsmit et al. 2018), along the southern Labrador coast in Mary's Harbor, St. Lewis, Charlottetown, and Cartwright (NL, Canada; 52°17′ N, 55°49′ W, 52°21′ N, 55°41′ W, 52°46′ N, 56°06′ W, 53°43′ N, 57°00′ W). However, we failed to detect the presence of any specimens.

### 2.2 Determination of Substrate Rugosity, Population Density, Snail Shell Height and Algal Biomass

Substrate rugosity, snail population density and shell height, as well as algal biomass, were measured, on site, at each sampling location. We haphazardly laid three, 10 m long transects, horizontal to the seashore (> 10 m distance between them) in the mid‐intertidal zone during low tide. For each transect, ten 0.25 m^2^ quadrats were placed at a distance of 0.5 m from one another to cover the entire transect length and assess snail population density, size structure and algal biomass. First, we measured substrate rugosity for each transect. Given that substrate rugosity can be measured using various methods such as profile gauges, stereophotography, and chains, which yield broadly comparable measurements (Frost et al. 2005), we decided to use a modified version of the chain method, providing a simple yet efficient approach. Specifically, we laid a vinyl measuring tape, carefully and tightly following the substrate roughness for the whole transect length. The ratio of the horizontal distance covered by the tape and the actual transect length (10 m) was used as a substrate rugosity proxy (Frost et al. 2005). To estimate population density, we counted all individuals inside the quadrats, and up to 1500 individuals *per* site were collected for shell height measurements. Using electronic calipers (Absolute IP‐67; Mitutoyo, Kawasaki, Japan; precision: ±0.01 mm) we measured the shell maximal height of all collected individuals (Figure S1). Snail shells were measured either on site or transported alive to the Maurice‐Lamontagne Institute (MLI), Fisheries and Oceans Canada (Mont Joli, QC, Canada; 48°38′ N, 68°09′ W), and the University of Quebec at Rimouski (Rimouski, QC, Canada; 48°27′ N, 68°30′ W) to be measured subsequently (Table S2). Finally, algal biomass was determined for each transect in three out of 10 haphazardly selected quadrats, by removing all macroalgae inside and weighing it using a portable kitchen scale (Starfrit, Longueuil, QC, Canada; precision: ±1 g), after removing excess water manually.

### 2.3 Determination of Environmental Factors

Based on their influence for intertidal ectotherms, we selected 10 environmental factors of potential impact for *L. littorea* to determine how they related to local population density and shell height (Meager et al. 2011; Hamilton and Gosselin 2020). The selected physical variables included: (1) summer (from June to September) mean sea surface temperature (SST), (2) 90th percentile of SST during the summer period, (3) 65th percentile of SST during the summer period, (4) maximum recorded SST, (5) growing season length as the number of days where daily mean SST exceeded 10°C, (6) mean SST for the spawning period (from April to July), (7) SST standard deviation (SD) for the summer period, (8) mean annual air temperature, (9) mean substrate rugosity, (10) mean algal biomass. We did not include salinity within the selected environmental factors because first, salinity is not considered a main driver of range dynamics in *L. littorea* (Goldsmit et al. 2018) and second, the mean sea surface salinity values vary little among locations and are close to oceanic values in the study area (30.7–32.1, see Table S3). Consequently, we did not expect to observe an effect on the traits we investigated (Lillebjerka et al. 2023). We obtained thermal data for the closest possible geographic position to each sampling site, respectively, from NASA's Multi‐scale Ultra‐high Resolution (MUR) SST product, accessed through the NOAA data server ERDDAP (Chin et al. 2017) and the *Terraclimate* dataset (Abatzoglou et al. 2018) for air temperature data, as intertidal species are influenced by both water and air temperature. We chose not to use a buffer of multiple geographic points to avoid the risk of including locations too far from the sampling sites. We utilized a period of 5 years (from 2017 to 2022) of thermal measurements to characterize the study locations over the species' generation time of 2–3 years and general life span of about 5 years (Moore 1937). Length of the growing season was included because of its importance for ectotherm growth and reproduction (Conover 1990; Conover and Present 1990; Bradshaw and Holzapfel 2008). The 10°C temperature threshold for growing season length was selected based on a previous study on *L. littorea* showing that temperatures around 10°C correspond to the species' maximum spawning (Chase and Thomas 1995). The spawning period of *L. littorea* was estimated based on previous observations of spawning timing for populations in New Brunswick (Chase and Thomas 1995). Air temperature was included in the analysis due to its consistently strong influence on intertidal communities (Morelissen and Harley 2007; Thyrring et al. 2017).

### 2.4 Distance Matrix Creation

To include the effect of purely spatial processes, we calculated the distance between sites and built a distance matrix based on non‐Euclidean distance by tracing the shortest over‐water path from one site to another. The matrix represents the pairwise spatial distances among all sites (Legendre 1993).

### 2.5 Statistical Analyses

To examine the effect of the environmental factors on *L. littorea* population density and shell height, we first determined the effect of spatial autocorrelation among sampling locations to avoid violations of the assumption of independence of observations (Legendre 1993). Thus, we performed a Mantel test using the distance matrix and environmental factors. The preliminary Mantel test showed a strong positive correlation between geographic distance and environmental differences among sampling locations (*r* = 0.69, *p* = 0.001). We therefore used a linear regression with latitude and longitude of the locations as factors, to remove spatial autocorrelation from environmental variables and used the residuals of the models in the subsequent analyses instead of the original factors' values.

To evaluate the effect of the selected “residualized” environmental factors on mean snail population density and shell height as well as to identify how they change following the environmental gradient covered by the study, we used linear regression analyses: for example, using the formula: *Density* ~ *Environmental factor 1* + *Environmental factor 2*. The 95th percentile of shell height, which best represents the upper growth limit of each population, was also analyzed to verify the consistency with observed trends for mean shell height. We then adopted the Akaike Information Criterion (AIC) with correction for small sample size (AICc) for model selection to determine the best‐fit environmental factor(s) for the model. For snail mean density, we compared models with one and two environmental factors; based on preliminary analyses, the best model within ∆AIC ≤ 2 was a model with a single factor; thus we did not include more complex models in the selection (Table S4). For snail mean shell height, a similar approach was used; although since the best model, with an ∆AIC ≤ 2 was a model with two factors, we also included models with one more factor (i.e., up to three factors) and a model with the interaction term in the selection process (Table S4).

To analyze the snail shell height structure at each location we divided shell heights into three size classes: *Small* (1–11 mm), *Medium* (> 11 to 18 mm), and *Large* (> 18 mm). The size class thresholds were based on previous data on *L. littorea* growth (Williams 1964), which suggested that maturity in the species is reached at around 12 mm, and that from about 19 mm growth proceeds only very slowly. We did not find evidence in the literature of *L. littorea* individuals reaching maturity at different shell heights depending on the location of origin. Hence, the selected size classes were considered to represent distinct maturation stages (Williams 1964; Saier 2000). It is important to note that the size class thresholds should be considered as only indicative of the actual developmental stages, as we did not directly verify gonadal maturation. However, Fish (1972) showed that snails from different locations (open coast versus estuarine) had comparable shell heights at maturity.

To include the entire set of selected environmental factors in the size structure analyses, we first performed a Principal Component Analysis (PCA) and extracted the PC1 and PC2 scores for each location. We then used a multinomial regression model, with PC1 and PC2 scores as fixed terms and the size classes as the response variable, using the *Medium* class as reference for the model.

All statistical analyses were done using R software, version 4.4.1 (R Core Team 2024, R Foundation for Statistical Computing, Vienna, Austria. <https://www.R‐project.org/>). For the PCA and Mantel test, we used the R package *vegan* (Version 2.6.8; Oksanen et al. 2024). For the size structure multinomial regression model, we used the R package *VGAM* (Version 1.1.13; Yee 2010). The statistical results were reported using the “language of evidence” guidelines proposed by Muff et al. (2022) to allow for a more refined and rigorous way to interpret the results. Briefly, evidence of effects is described following this range: 0.0001 < *p* < 0.001—very strong evidence; 0.001 < *p* < 0.01—strong evidence; 0.01 < *p* < 0.05—moderate evidence; 0.05 < *p* < 0.1—weak evidence; 0.1 < *p* < 1—little to no evidence.

## 3 Results

### 3.1 Population Density

**FIGURE 2:**
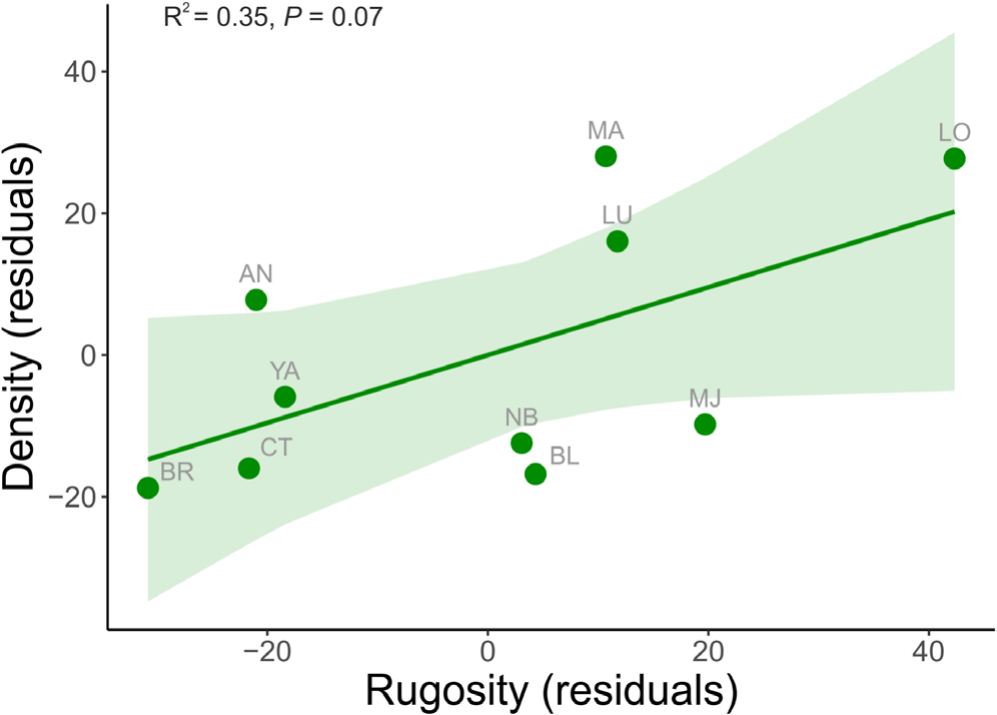
Relationship between substrate rugosity and *Littorina littorea* density measured in different locations across the species' geographical distribution range on the Atlantic coast of North America. The dots, line and shaded area correspond to a location‐averaged data point, the regression line and the 95% confidence interval of model predictions, respectively. *R* ^2^ and *p* values are also reported in the figure.

Snail mean density ranged from 37 (CT—the southernmost collection site) to 235 (LO—approx. at the mid‐range of the latitudinal gradient investigated) individuals m^−2^. Among the selected environmental factors, substrate rugosity showed the best fit for snail density (Table S4) with a weak positive linear relationship between snail density and rugosity (*R* ^2^ = 0.35, *β* = 0.48, *t*(8) = 2.07 *p* = 0.07, Figure 2).

### 3.2 Snail Shell Height

**FIGURE 3:**
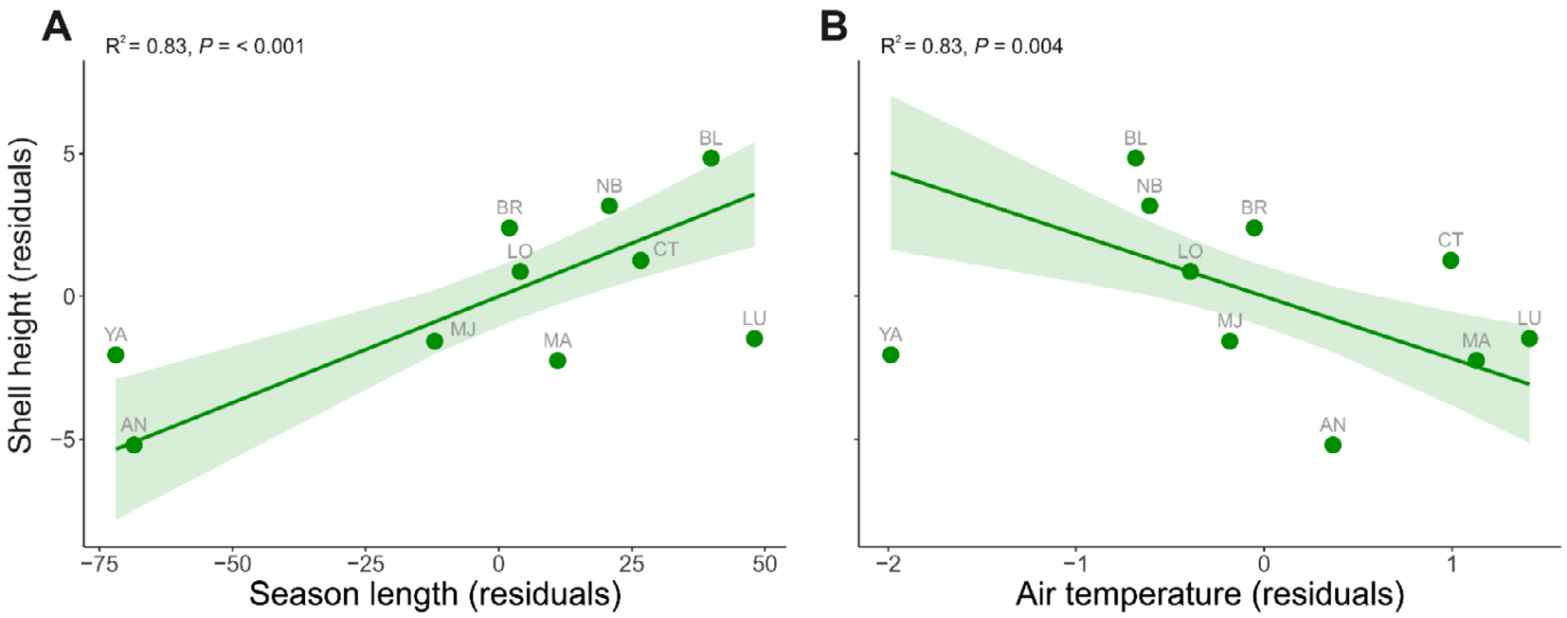
Relationship between *Littorina littorea* mean shell height and (A) season length, (B) mean air temperature measured or extracted in different locations across the species geographical distribution range on the Atlantic coast of North America. The dots, line, and shaded area correspond to a location‐averaged data point, the regression line, and the 95% confidence interval of the models' predictions, respectively. *R* ^2^ and *p* values are reported in the figure.

Mean snail shell height ranged from 9.7 (AN) to 20.3 (NB) mm. Among the selected environmental factors, season length and the air temperature at the sampled locations best predicted snail shell height (Table S4). Our model indicated very strong evidence (*R* ^2^ = 0.83, *β* = 0.075, *t*(7) = 5.67, *p* < 0.001) of snail shell height increasing linearly with increasing season length (Figure 3A) and strong evidence (*R* ^2^ = 0.83, *β* = −2.18, *t*(7) = −4.12, *p* = 0.004) of snail shell height decreasing linearly with a warmer air temperature (Figure 3B). The model using the 95th percentile of shell height yielded results nearly identical to those obtained with mean shell height (Figure S2).

### 3.3 Snail Size Structure

**FIGURE 4:**
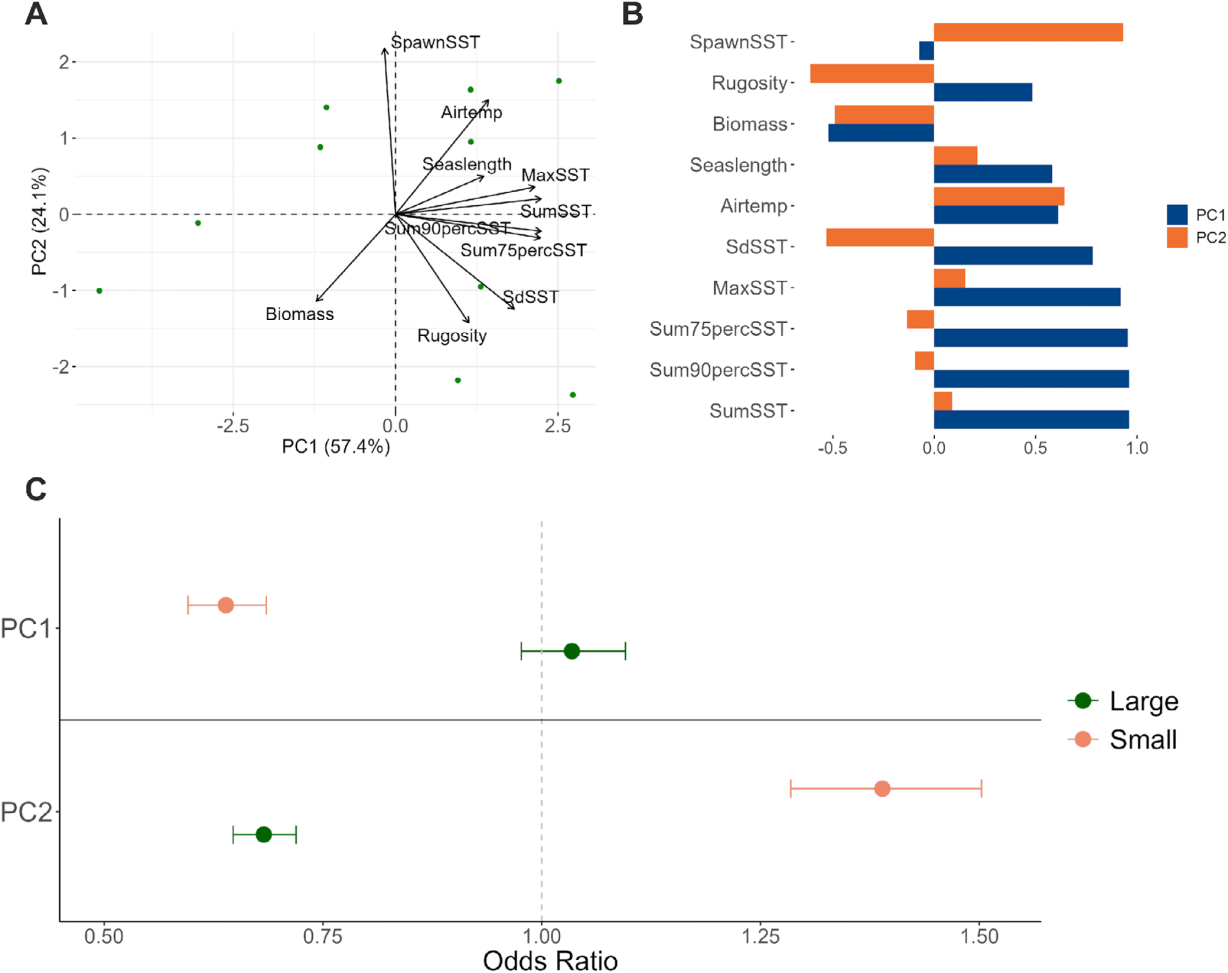
Results of *Littorina littorea* size structure analysis evaluating the relationship between a locations' environmental factors and *Large* and *Small* size class frequencies. (A) PCA biplot representing environmental factors' contributions (arrows) on the first two principal components (PC1 and PC2) and locations' data points (dots, green for *Large* and orange for *Small*). (B) Individual factor positive and negative contributions to PC1 (blue) and PC2 (orange). (C) Relationship between PC1 and PC2 on the *Large* and *Small* size class frequencies compared to the *Medium* class. Whiskers indicate the 95% confidence intervals (CI) of the odds ratio estimation; if the CI fell within the 1 ratio threshold (indicated by the dotted line), the PC relationship with the size class was not considered significant.

The first two principal components (PC) scores together explained 81% (PC1 = 57%, PC2 = 24%) of the total variation among the environmental factors for our 10 study sites (Figure 4A,B). The environmental factors with the largest contribution to PC1 were *Summer SST*, *Season Length*, *Max SST*, *Summer 75th percentile SST*, *Summer 90th percentile SST* and *Summer SD SST* (Figure 4B). The environmental factor with the largest contribution to PC2 was *Spawning period SST* (Figure 4B). It is worth noting that *Rugosity* and *Summer SD SST* had a positive contribution to PC1 and a negative contribution to PC2 (Figure 4B).

Our multinomial regression model showed a negative relationship between PC1 and the *small* size class frequency (Figure 4B), indicating that with an increase in PC1 there was a decrease in the frequency of small size snails, compared to medium size ones. Conversely, there was no evidence of a significant relationship between PC1 and the *large* size class frequency (Figure 4B). Our model showed that PC2 had a positive relationship with the *small* size class frequency and a negative relationship with the *large* size class frequency. This result indicated that, compared to the *medium* size class, when PC2 increased the small size snail frequency increased, whereas large size snail frequency decreased.

## 4 Discussion

Our results provide clear evidence of the presence of clinal variation in mean population density, mean shell height, and size structure along the non‐native geographic range of distribution in Atlantic North America of the intertidal gastropod *L. littorea* . Intriguingly, the variations in mean density and mean shell height are mainly influenced by different environmental aspects of the locations of origin. Density variation along the gradient is best explained by differences in local conditions, specifically substrate rugosity, which relates positively to density. Inversely, the variation in shell height is best explained by the length of the growing season and air temperature, which positively and negatively affect mean shell height, respectively. In addition, higher and more variable summer water temperatures are related to a decreased frequency of small individuals, whereas warmer water temperatures during the spawning season coincide with increased frequency of small individuals and a decrease in the frequency of large ones. These findings highlight the importance of considering multiple environmental factors to explain latitudinal macroecological patterns in abundance and size along broad geographical ranges.

### 4.1 Local Substrate and Climate Best Explain Variation in Mean Density and Shell Height, Respectively

Our study showed that for *L. littorea* , more complex substrates increased the density of individuals. This is not surprising, as complexity is associated with an increase in sheltered areas represented by cracks, boulders and crevices, and in general with a higher surface area for intertidal benthic fauna to settle (Kohn and Leviten 1976; Carlson et al. 2006; Suzanne Witte et al. 2025). It is of note that we expected temperature to also have a strong impact on snail density given the important role it plays on species abundance in rocky intertidal habitats (Thyrring et al. 2017; Amstutz et al. 2021). That substrate rugosity has the best fit suggests the important role habitat complexity plays in defining abundance, to the point of outweighing the effects of temperature for *L. littorea* .

In contrast to density, mean shell height was primarily affected by climate. The positive relationship between season length and mean shell height may reflect the thermodynamic effect of temperature on growth rates, with higher temperatures accelerating metabolic processes. Alternatively, this relationship may result from a longer duration of favorable thermal conditions that simply extends the time available for growth (Brown et al. 2004).

An important observation concerning mean shell height of snails in relation to a location's air temperature is that, as observed in other intertidal ectotherms (Lee and Boulding 2010; Hernández‐P et al. 2023), *L. littorea* conforms to Bergmann's rule, with larger individuals found in cold locations. This pattern is mechanistically explained as the result of physiological constraints and compensation mechanisms (Levinton 1983). However, in *L. littorea* , the increase in shell height with colder air temperatures may be explained by various mechanisms. First, colder air temperatures are related to slower metabolism, thus sexual maturity may be delayed and occur at larger shell heights relative to hotter conditions, leading to a larger overall mean shell height (Atkinson and Sibly 1997). Second, smaller body size has been related to better thermal tolerance under warmer conditions in aquatic ectotherms (Daufresne et al. 2009), with factors, such as reduced oxygen limitation in smaller individuals, proposed to explain this relationship (Leiva et al. 2019). Furthermore, a negative relationship between shell height and upper thermal tolerance has previously been demonstrated in *L. littorea* (Clarke et al. 2000), and more recently confirmed in individuals from the same locations examined in the present study (Garlaschè et al. 2025, in press) suggesting larger individuals may be less tolerant.

### 4.2 Multiple Environmental Factors Jointly Shape Size Structure Along the Gradient

The observed geographic size structure may reflect heat stress‐induced mortality during early small stages, caused by higher and more variable summer temperatures, reducing the proportion of small individuals in populations (Truebano et al. 2018). In contrast, warmer spawning periods shift the size structure of *L. littorea* toward smaller individuals, suggesting that warm temperatures from April to July may favor recruitment, hatching success, and/or juvenile survival. The negative effect of substrate rugosity on the frequency of small snails may be linked to the association between habitat complexity and invertebrate body size reported in other studies (Meager et al. 2011), although the mechanisms underpinning the positive relationship we report in *L. littorea* remain elusive.

### 4.3 Climatic Influence on Population Dynamics in Rocky Shores

Together, our results suggest that a longer growing season, higher and more variable water temperatures in the summer lead to an increase in snail mean shell height and a decrease in the frequency of small snails, respectively. Likewise, we found evidence that air temperature has a negative effect on mean shell height but does not seem to alter population size structure.

While elevated temperatures may enhance growth rates, our results support the notion that heat stress may increase juvenile mortality. Notably, season length was a stronger predictor of shell height rather than summer temperature, indicating that longer growing windows, rather than warmer conditions, are the main drivers of the increase in mean shell height. In line with this interpretation, physiological measures obtained from *L. littorea* individuals collected in the same sampling area and time (Garlaschè et al. 2025, in press) indicated that snails from warmer locations do not possess an overall greater growth rate. In the context of ocean warming, warm‐adapted populations are expected to be more vulnerable to further increases in temperature, as their upper thermal limits are already close to their environmental thermal maxima, and their acclimation capacity may be limited (Stillman 2004; Somero 2010; Sunday et al. 2012). Physiological evidence has shown that in the closely related *Littorina saxatilis* , southern individuals exhibit lower heat tolerance compared to their northern counterparts (Dwane et al. 2023; Titmuss et al. 2025). Furthermore, thermal performance curves of *L. littorea* from the same locations of our study indicated that snails from warmer locations experienced higher mortality and lacked acclimation capacity when exposed to high temperatures, compared to snails from colder locations (Garlaschè et al. 2025, in press). In this context, elevated juvenile mortality due to heat stress is a well‐documented phenomenon in intertidal species and may explain the observed decline in small snails with increasing summer temperatures (Moran 1999; Nasrolahi et al. 2013). This explanation aligns well with the range shift that *L. littorea* is experiencing in Europe with a progressive reduction in abundance toward the warm edge of the range and local extinction along the Iberian Peninsula (Rubal et al. 2013).

Another important finding was the positive influence of the spawning period temperature on the frequency of small individuals. This pattern is supported by evidence that warmer temperatures during the spawning period can prolong spawning duration and improve *L. littorea* hatching success (Chase and Thomas 1995; Lillebjerka et al. 2023), potentially promoting higher recruitment rates. Enhanced spawning may result in an increased proportion of juvenile snails, which is indicative of a potentially growing population (Gaines and Roughgarden 1985; Jeffrey 2003; Takada 2008). We also show that less variable summer temperatures have a positive effect on the frequency of small snails, which aligns well with the hypothesis of an enhanced juvenile survival rate. In particular, strong fluctuations in temperature can be deleterious for juvenile survival in intertidal gastropods (Truebano et al. 2018; Hamilton and Gosselin 2020).

## 5 Conclusion

Collectively, our findings support the conclusion that local conditions, such as substrate complexity, and environmental gradients, including the growing season length, air temperature, summer and spawning water temperatures, may differentially influence the population dynamics of an intertidal non‐native species.

In particular, given its influence on abundance, habitat complexity should be taken into account when attempting to identify current and potential future hotspots for an intertidal invasive species expanding its range. Furthermore, our considerations about mean shell height and size structure patterns provide an interesting perspective on possible future range shifts of *L. littorea* . As climate change progresses, the projected poleward shifts in suitable habitat for this species suggest it will encounter increasingly warmer conditions during its spawning period (Walsh et al. 2011). At the same time, Arctic summer temperatures are cooler than those in the studied locations, potentially reducing heat‐induced mortality and supporting population growth during range expansion (Bradley et al. 2024). In summary, our study supports the idea that *L. littorea* can take advantage of future conditions in the Arctic to expand its range northward, as suggested by Goldsmit et al. (2018, 2019), as well as suggesting that the species may reduce its abundance and potentially face local extinction at the southern distribution edge in the future.

The absence of individuals during our survey of the southern Labrador coast suggests that the species has not yet become established in the region, possibly due to extant currents preventing larvae from reaching these locations (Hohenlohe 2004). Nonetheless, the possibilities of anthropogenic dispersion to suitable areas in the Arctic are still high, given the ongoing increase of maritime traffic facilitated by climate change and driven by growing tourism and rich exploitable resources in the region (Ricciardi et al. 2017).

While our study emphasizes abiotic drivers of *L. littorea* population dynamics, we recognize that biotic factors may also influence these patterns. In particular, competition, predation, parasitism and intertidal vertical migration may play a role in the observed trends (Matassa and Trussell 2015; Granovitch 2016; Hargreaves 2024). Future studies could use biotic interactions along latitudinal gradients to delineate their impact on population dynamics. In our case, we chose to focus on abiotic factors to avoid excessive complexity in interpretation and to prioritize broad sampling coverage for capturing variation along the extensive *L. littorea* latitudinal gradient investigated. Future studies could also examine the interplay between environmental gradients and body temperature, and how this influences abundance and size, as microclimatic conditions in intertidal habitats play an important role in invertebrate thermal biology (Helmuth et al. 2006; Bozinovic et al. 2011).

Our findings provide valuable insights into large‐scale biogeographical patterns of range dynamics, response to environmental changes, and heat stress, for intertidal species. Incorporating a wide range of environmental factors to explain variation in species population dynamics offers a more nuanced and comprehensive perspective than relying solely on latitude. We highlight the importance of considering multiple environmental factors along latitudinal gradients to gain a comprehensive understanding of the drivers causing changes in population dynamics that ultimately help define a species biogeography.

## Author Contributions

**Giuseppe Garlaschè:** conceptualization (lead), data curation (lead), formal analysis (lead), investigation (lead), methodology (lead), project administration (lead), resources (equal), software (lead), validation (equal), visualization (lead), writing – original draft (lead), writing – review and editing (equal). **Bernardo R. Broitman:** conceptualization (supporting), data curation (supporting), formal analysis (equal), methodology (equal), software (supporting), supervision (supporting), validation (equal), visualization (equal), writing – original draft (supporting), writing – review and editing (equal). **Cyrena Riley:** conceptualization (supporting), investigation (equal), methodology (supporting), project administration (equal), resources (supporting), supervision (supporting), validation (supporting), writing – review and editing (equal). **David Drolet:** conceptualization (equal), funding acquisition (supporting), investigation (supporting), methodology (supporting), project administration (equal), resources (equal), supervision (supporting), validation (supporting). **Kimberly L. Howland:** conceptualization (supporting), funding acquisition (equal), investigation (supporting), methodology (supporting), project administration (supporting), resources (supporting), supervision (supporting), validation (supporting), writing – review and editing (equal). **Christopher W. McKindsey:** conceptualization (equal), funding acquisition (equal), investigation (supporting), methodology (supporting), project administration (equal), resources (equal), supervision (supporting), validation (supporting), writing – review and editing (equal). **Piero Calosi:** conceptualization (equal), formal analysis (supporting), funding acquisition (supporting), investigation (supporting), methodology (supporting), project administration (equal), resources (supporting), supervision (equal), validation (equal), visualization (equal), writing – original draft (supporting), writing – review and editing (equal).

## Conflicts of Interest

The authors declare no conflicts of interest.

## Supporting information


**Appendix S1:** ece372532‐sup‐0001‐AppendixS1.docx.

## Data Availability

The data and code that support the findings of this study are openly available on Dryad at https://doi.org/10.5061/dryad.d2547d8fh.
