# Supplementary material for: Local Conditions and Environmental Gradients Shape the Abundance and Size Structure of a Non‐Native Intertidal Species in Atlantic North America
Source: Ecol Evol. 2025 Dec 12;15(12):e72532. doi: 10.1002/ece3.72532 (PMC12699205; doi:10.1002/ece3.72532)
Supplement: Supplementary file 1 — Appendix S1: ece372532‐sup‐0001‐AppendixS1.docx. [file ECE3-15-e72532-s001.docx]

**Supporting information**


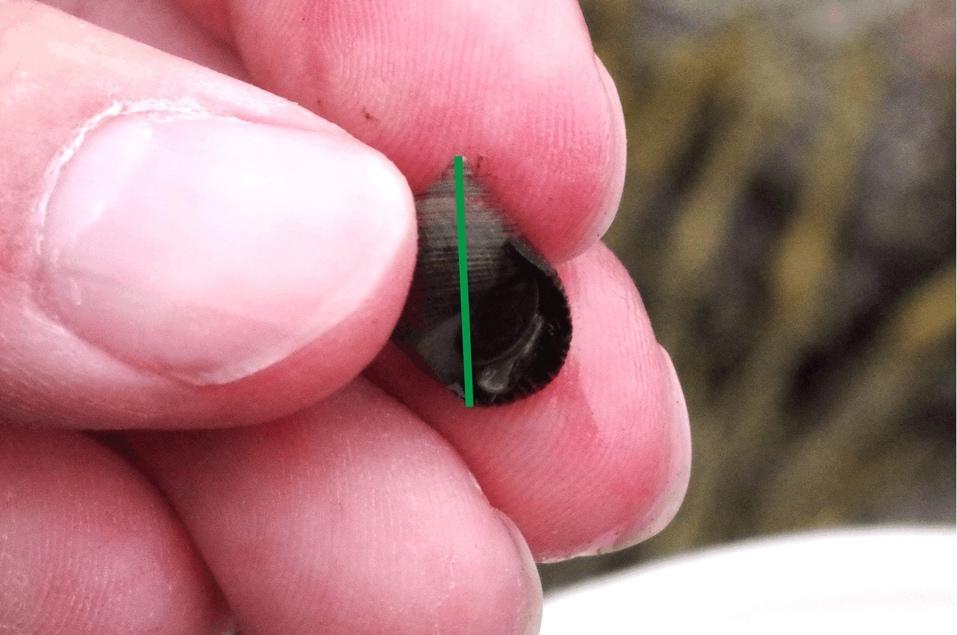


**Figure S1:** Picture showing how the maximal shell height (green line) was measured and used as a proxy for body size of *Littorina littorea*, by means of electronic callipers.

**Table S1:** Sampling locations with geographic coordinates and date of sampling.

| **Location** | **Code** | **Latitude (°N)** | **Longitude (°W)** | **Sampling date** |
| --- | --- | --- | --- | --- |
| Esker Point Beach | CT | 41.32 | 72.00 | 18/08/2022 |
| Magnolia Rocks | MA | 42.57 | 70.71 | 22/08/2022 |
| Lower East Pubnico | YA | 43.58 | 65.78 | 21/07/2022 |
| Marie Joseph Park | MJ | 44.97 | 62.09 | 23/07/2022 |
| Lorneville Breakwater | NB | 45.19 | 66.15 | 24/08/2022 |
| Louisburg | LO | 45.91 | 59.97 | 25/07/2022 |
| Branch | BR | 46.88 | 53.95 | 28/07/2022 |
| Lumsden Harbour | LU | 49.32 | 53.61 | 30/07/2022 |
| Blanc Sablon | BL | 51.41 | 57.20 | 08/08/2022 |
| Anse aux Meadows | AN | 51.60 | 55.54 | 02/08/2022 |

**Table S2:** Total number of *Littorina littorea* individuals measured to obtain the body size data at each sample location. See Figure 1 for the full spelling of location abbreviations.

| **Location** | **Measured *in situ*** | **Measured at IML** | **Measured at UQAR** | **Total of individuals** |
| --- | --- | --- | --- | --- |
| CT | 100 | 175 | 0 | 275 |
| MA | 150 | 1358 | 0 | 1508 |
| YA | 656 | 0 | 0 | 656 |
| MJ | 723 | 0 | 0 | 723 |
| NB | 0 | 487 | 0 | 487 |
| LO | 345 | 0 | 1000 | 1345 |
| BR | 428 | 0 | 0 | 428 |
| LU | 428 | 0 | 0 | 428 |
| BL | 458 | 0 | 0 | 458 |
| AN | 501 | 0 | 645 | 1146 |

**Table S3:** Salinity values of the sampled locations, obtained from Copernicus Marine Service website (<https://data.marine.copernicus.eu/product/MULTIOBS_GLO_PHY_S_SURFACE_MYNRT_015_013/description?utm_source=chatgpt.com>). Mean, minimum and maximum values are based on daily salinity data (expressed as PSU) for the period 1993-2025.

| Location | Mean Salinity | Min. Salinity | Max. Salinity |
| --- | --- | --- | --- |
| CT | 31.3 | 26.7 | 34.7 |
| MA | 31.8 | 26.9 | 31.8 |
| YA | 31.6 | 28.3 | 34.1 |
| NB | 31.2 | 26.9 | 34.2 |
| MJ | 30.8 | 28.2 | 32.9 |
| LO | 30.7 | 27.6 | 32.5 |
| BR | 31.8 | 29.6 | 33.1 |
| LU | 32 | 29.3 | 33.6 |
| BL | 31.5 | 27.5 | 33.6 |
| AN | 32.1 | 27.8 | 34 |

**Table S4:** Summary of the results of the multiple linear regression models comparisons using Akaike Information Criterion for small sample sizes (AICc) scores. Only the best 10 models are shown. The models in bold represent those finally selected for the analysis for mean population density and mean body size.

| **Variables** | **AICc** | **∆AICc** | **R^2^** |
| --- | --- | --- | --- |
|  |  |  |  |
| **Density** *versus*: |  |  |  |
| **Rugosity** | **91.269** | **0.000** | **0.350** |
| Rugosity + Biomass | 93.997 | 2.729 | 0.531 |
| Airtemp | 94.023 | 2.755 | 0.143 |
| Biomass | 94.159 | 2.890 | 0.132 |
| Sum90percSST | 94.687 | 3.418 | 0.085 |
| MaxSST | 94.839 | 3.571 | 0.070 |
| Sum75percSST | 94.981 | 3.712 | 0.057 |
| SumSST | 95.043 | 3.774 | 0.051 |
| SdSST | 95.061 | 3.792 | 0.050 |
| Rugosity + Airtemp | 95.266 | 3.998 | 0.468 |
|  |  |  |  |
| **Size** *versus*: |  |  |  |
| **Seaslength + Airtemp** | **47.69** | **0.00** | **0.83** |
| Seaslength + Airtemp + Rugosity | 51.21 | 3.52 | 0.90 |
| Seaslength * Airtemp | 53.53 | 5.83 | 0.88 |
| Seaslength | 54.02 | 6.33 | 0.42 |
| Seaslength + Airtemp + Biomass | 54.75 | 7.06 | 0.86 |
| Seaslength + Airtemp + SpawnSST | 56.04 | 8.35 | 0.84 |
| Seaslength + Airtemp + MaxSST | 56.15 | 8.46 | 0.84 |
| Seaslength + Airtemp + SdSST | 56.56 | 8.87 | 0.83 |
| Seaslength + Airtemp + SumSST | 56.59 | 8.90 | 0.83 |
| Seaslength + Airtemp + Sum75percSST | 56.68 | 8.99 | 0.83 |


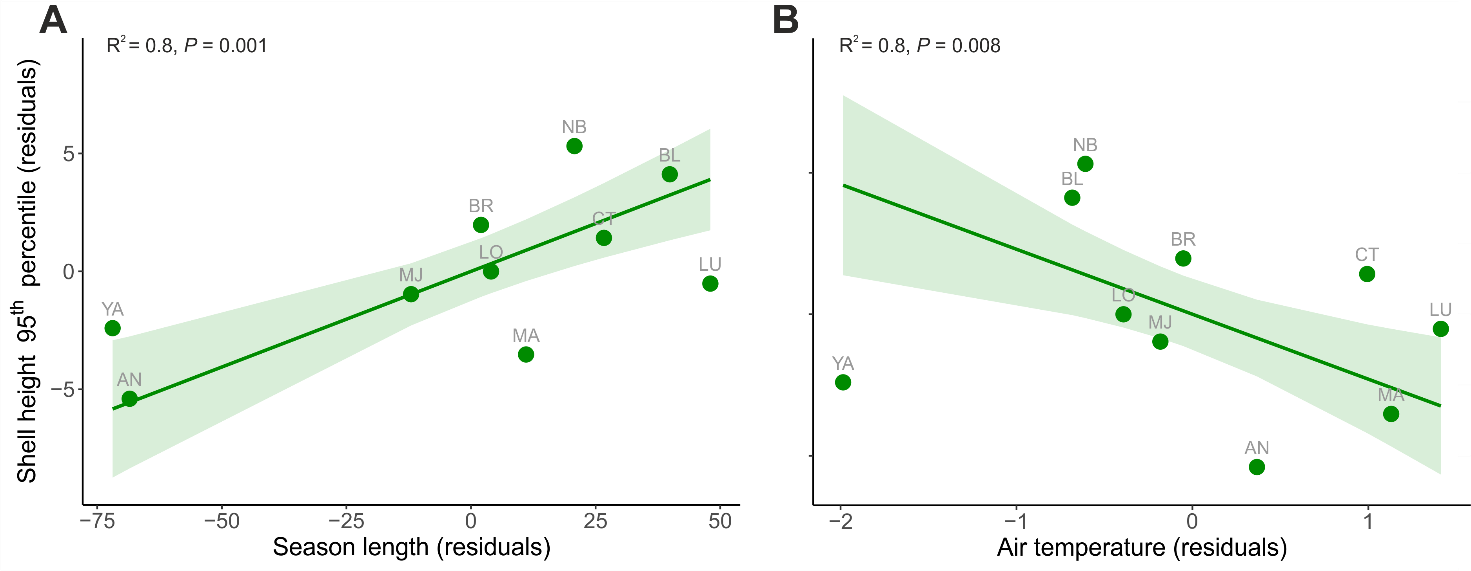


**Figure S2:** Relationship between *L. littorea* 95^th^ percentile shell height and (A) season length, (B) mean air temperature, measured or extracted in different locations across the species geographical distribution range on the Atlantic coast of North America. The dots, line, and shaded area correspond to a location data point, the regression line, and the 95% confidence interval of the models’ predictions, respectively. R^2^ and *P* values are reported in the figure.
